# Supplementary material for: Are carbon emissions trading and green financial instruments synergistic? -Comprehensive quantitative research based on content analysis
Source: PLoS One. 2024 Mar 7;19(3):e0298601. doi: 10.1371/journal.pone.0298601 (PMC10919601; doi:10.1371/journal.pone.0298601)
Supplement: S1 Data — (ZIP) [file pone.0298601.s001.zip › Data/地级市Z_F工作报告.zip!/╣≤╓▌/░▓╦│╩╨/└┤╫╘╣1⁄2╓┌║┼í╛┬φ┐╦╩2╛▌═°í┐_2018.docx]

各位代表：

现在，我代表市人民政府向大会作工作报告，请予审议，并请市政协委员和其他列席会议的同志提出意见。

一、2017年工作回顾

刚刚过去的2017年，是本届政府履职的开局之年，我们在省委、省政府和市委的坚强领导下，在市人大、市政协的监督、支持下，团结和带领全市各族人民，深入学习贯彻党的十八大、十八届历次全会、党的十九大和习近平总书记系列重要讲话精神，认真贯彻落实省第十二次党代会和市第四次党代会精神，按照“守底线、走新路、奔小康”的工作总纲，坚持主基调主战略，以“新型城镇化+”为主线，扎实推进大扶贫、大数据、大生态三大战略行动，持续打好“一二四”攻坚战，凝心聚力、苦干实干，经济社会保持快速健康发展。

全市实现地区生产总值802.46亿元，增长12.3%；500万元以上固定资产投资794.54亿元，增长21.7%；规模以上工业增加值214.68亿元，增长12.1%；社会消费品零售总额197.11亿元，增长11.8%；一般公共预算收入76.24亿元，增长12.3%；金融机构存贷款余额1173.14亿元和812.39亿元，分别增长7.5%、18.1%；城镇和农村居民人均可支配收入27224元和8956元，分别增长9.4%、10.3%；居民消费价格上涨0.7%。

一年来，我们主要做了以下工作。

（一）坚持抓项目强招商，固定资产投资取得新成效

项目建设加快推进。我们牢牢抓住项目建设这个“牛鼻子”，推行市级领导联系挂帮制度和“三集中、三倒逼”工作机制，先后组织6次重大工程和重点项目集中开工，开工项目284个，实施重大工程和重点项目544个，完成投资1005亿元。组织7次PPP项目集中开工，实施项目29个，总投资404.7亿元。投资结构不断优化，第三产业投资增长27.2%，占比提升9.95个百分点。

发展平台不断夯实。我们牢牢抓住发展平台这个强力支撑，坚持不懈抓创建，锐意创新促发展。安顺高新区成功升格国家级高新区，列入全省“1+8”开放平台。我市获批列入全国首批通用航空产业综合示范区、第二批国家级旅游改革创新先行区、全国第二批生态修复城市修补试点，成功创建国家卫生城市、国家园林城市，获评中国最佳避暑旅游城市。安顺经开区获批“国家火炬安顺航空智能制造特色产业基地”。

招强引优成效明显。我们牢牢抓住招商引资这个重要推手，大力实施“千企引进”工程，广泛开展各类招商活动。先后赴北京、上海、重庆、深圳等地开展资本市场助推脱贫攻坚、军民融合通用航空、大数据智能终端、旅游产业等大型招商推介活动，积极参加“贵洽会”“数博会”“渝洽会”等系列招商活动。引进复星集团、希望集团、特变电工等500强企业来安投资，酷骑、沃客、汤姆逊等37家大数据智能终端及配套企业落户我市。全年新引进省外3000万元以上项目517个，到位资金1164.27亿元，增长29.6%。

（二）坚持抓质量提效益，产业转型升级取得新进展

现代农业稳步发展。以农业供给侧结构性改革引领现代农业发展，结构调整成效明显。蔬菜、食用菌、茶叶、精品水果、中药材、生态畜禽等特色产业规模不断扩大，畜牧业产值占一产比重继续保持全省领先，绿色农产品实现“泉涌”发展。农业园区加快建设，新增6个省级农业园区，乡镇农业园区效益不断提升。新型农业经营主体发展壮大，新增市级以上龙头企业14个、农民专业合作社923个、家庭农场28个。农产品质量安全水平稳步提升，“三品一标”认证数达155个。加工销售和市场开拓取得新进展，农产品加工企业达857家，农产品加工率达72.4%，农村电商综合服务站达500个，在山东、内蒙古、长三角、珠三角等地开展特色农产品展销推介，品牌知名度不断提升。

新型工业持续发力。以工业经济提质增效为目标，聚焦传统产业稳产增效和新兴产业培育壮大。军民融合产业加快发展，贵飞工业联合体初步构建，自主研制生产的山鹰首架外贸飞机总装下线，安大航空锻造产业园、安吉精铸航空产业园加快建设，风雷氧业公司建成投产，与省科技厅组建安捷航宇公司，成功举办爱飞客通用航空飞行大会。大数据电子信息产业聚集发展，西秀智能终端产业园加快建设，西部生态智谷项目积极推进。特色产业稳步发展，中国（安顺）石材城建成投用，成功举办第三届石博会，晨春石材、天韵石尚等石材企业不断壮大。实施“千企改造”工程重点项目100个，煤、电、铝等传统产业转型升级步伐加快，白酒、制药、食品等特色轻工产业链不断延伸，黄果树金叶科技烟叶综合利用项目全面建成投产。

服务业发展提档升级。以创建国家全域旅游示范区为目标，旅游基础设施和配套服务不断完善，旅游与其他产业不断融合，全域旅游持续深化。推出奇缘谷冰雪小镇、九龙山森林公园休闲度假区等一批旅游产品，虹山湖被评为国家4A级旅游景区，黄果树智慧旅游公司在新三板挂牌上市，成功举办2017环中国国际公路自行车赛等赛事活动，旅游业持续“井喷”，全年接待游客7300万人次，增长35%，实现旅游收入750亿元，增长38%。大健康产业加快发展，全市大健康医药产业实现增加值120亿元，增长20%。黄铺新区建设加快推进，大兴东产业园、亿丰国际物流园入选省级现代服务业集聚区。全年实现服务业增加值400亿元，增长15%。

（三）坚持抓试点促融合，城乡建设取得新突破

试点工作成效明显。抢抓试点工作机遇，加快推进“五型城市”建设。“一分三向”新型城镇化安顺模式深入实施，农业转移人口市民化成本分担机制初步形成，户籍人口城镇化率达38%，常住人口城镇化率达51%，顺利通过国家新型城镇化综合试点中期评估。“多规融合”试点工作取得新进展，全市土地利用总体规划调整方案获省政府批复，城乡空间管控体系基本划定。园区与城镇实现互动发展，镇村联动有序推进。

基础设施日益完善。综合交通建设加快推进，黄果树机场改扩建工程和乐平通用机场启动建设，安六铁路建设进展顺利，现代有轨电车3、4、6号线前期工作有序推进，贵安大道（经开区至黄果树段）、关花大道、平坝东外环线、安宁大道等骨干道路建设加快推进，花安高速、安紫高速、黄铺大道建成通车，完成139公里国省道干线改造。水利建设顺利推进，黄家湾大型水利枢纽工程加快建设，坝陵河、木桥中型水库开工建设，芦坝、雨棚、打冲沟、白沙、小鲁灰、龙宫河等骨干水源工程快速推进，新场河、老营水库主体完工，猫洞河、纳井田水库下闸蓄水，新增有效灌溉面积2.6万亩，解决农村饮水安全21万人。完成电力基础设施投资7亿元，新一轮农村电网改造扎实推进，新建、改造10千伏及以下线路3736公里。完成信息基础设施投资10亿元，实现全市自然村光网全覆盖，3G/LTE基站总数达1万个，城域网上链带宽达500Gbps以上。

城乡建设步伐加快。城市路网进一步完善，贯城河滨水景观综合治理一期工程基本完成，历史文化街区改造全面启动，安顺高铁新城、虹山湖新区、平坝高铁新区初具规模，普定、镇宁、关岭、紫云县城活力不断增强，建成9个城市综合体。35个示范小城镇带动能力不断提升，黄果树镇列入全国特色小镇名录，丁旗石材小镇、黎阳航空小镇雏形初现。六项小康行动计划扎实推进，农村人居环境持续改善，建成马鞍山、节溪、猛邦等20余个美丽乡村。

生态建设稳步推进。“绿色贵州”建设三年行动计划全面完成，实施营造林72.7万亩，综合治理石漠化面积128平方公里，治理水土流失面积220平方公里，森林覆盖率达54% 。黄果树湿地公园开放运营，邢江河、虹山湖湿地公园加快建设。全面开展河长制工作，实行最严格水资源三条红线管理，饮用水源地水质达标率100%。县级以上城市污水处理率达92.4%，城乡生活垃圾无害化处理率达88%，中心城区环境空气质量优良率达96.7%。单位地区生产总值能耗下降3.2%，碳强度下降3.6%。

（四）坚持抓重点补短板，脱贫攻坚取得新胜利

精准扶贫扎实有效。以脱贫攻坚统领经济社会发展全局，出台《安顺市深度贫困地区脱贫攻坚行动实施方案》等“1+N”政策措施，全力抓好脱贫攻坚春季攻势、夏季大比武、秋季攻势，打好“四场硬仗”“七大战役”。全面启动农村“组组通”公路三年大决战，建成通组公路1184公里。省部共建石漠化片区水利精准扶贫示范区建设有序推进，61个示范项目实施方案获批。资助14648名高中以上农村建档立卡贫困学生，免除8371名困难家庭高中学生学费。推进“四重医疗保障”制度落实，实行县域内农村贫困人口“先诊疗后付费”救治、医疗保障“一站式”即时结报，补偿55219人次，实际报销补偿比达90.2%。社会帮扶扎实推进，“千企帮千村”行动深入开展。全年减少农村贫困人口10.55万人，贫困发生率下降到7.55%。

产业脱贫成效显著。紧紧抓住产业脱贫这个主攻方向，蔬菜、食用菌、金刺梨、火龙果、红芯薯、关岭牛、紫云花猪等发展迅速。97个扶贫产业子基金项目通过评审，获批基金55.4亿元。发放“特惠贷”6.03亿元，财政贴息3020.6万元。加快全市178个深度贫困村脱贫步伐，每村安排100万元引导基金发展扶贫产业。产业扶贫利益联结机制不断完善，涌现出西秀“菜单式”扶贫、普定“一村一公司”、关岭“五户联保”、经开区食用菌产业带动等典型模式。

易地扶贫搬迁深入推进。聚焦打好易地扶贫搬迁关键一仗，2016年度7239户29792人全部实现搬迁入住。加快推进2017年度7446户33533人搬迁工作，西秀鸡场乡，普定鑫旺大市场、玉兔山，镇宁简嘎乡及关岭顶云新城、岗乌镇6个安置点提前建成并搬迁入住，其余的8个安置点实现主体完工。

（五）坚持抓改革求突破，发展动力取得新升级

重点改革进展顺利。聚焦重点、难点、痛点，以改革释放动力活力。推进供给侧结构性改革，严格落实“三去一降一补”重点任务。创新金融产品，通过“微保贷”“中小企业创业贷”等帮助企业融资近亿元。城市管理综合执法体制改革有序推进。深化农村综合改革，夯实“三权”促“三变”基础，“七权”同确试点和不动产登记工作有序推进，“塘约经验”在全市75%的行政村中得到推广，全市村级集体经济达11.9亿元。平坝区被确定为全国农村集体产权制度改革试点，普定县被确定为全国自然资源统一确权登记工作试点，安顺经开区被确定为首批全国农村社区治理实验区。

市场活力显著提高。推动大众创业、万众创新，新经济、新产业、新业态不断涌现。深化商事制度改革，全面推进“多证合一”，扶持发展微型企业1850户，各类市场主体突破15万户。建成省级工程技术研究中心3个、市级工程技术研究中心10个、市级众创空间3个，新增国家高新技术企业6户。民营经济不断发展，民营经济增加值占生产总值比重达65.5%以上。

对外开放逐步扩大。以国家新型城镇化综合试点为引领的“1+5”开放平台建设扎实推进。青岛安顺扶贫协作持续深化，教育、医疗、旅游等方面合作不断深入，两地合拍的《安顺故事》荣获亚洲微电影最高奖项“金海棠奖”，开通安顺至青岛航线，青安产业园建设有序推进，青岛榕昕集团等企业落户安顺。与贵安新区“五联十同”项目积极开展，贵阳—贵安—安顺同城化进程加快推进。与港澳台、欧盟、东盟等地交流合作不断加强。安顺公用型保税仓库投入运营。全市外贸进出口总额2亿美元，实际利用外资2.04亿美元。

（六）坚持抓基础促民生，社会事业取得新进步

民生保障持续改善。全市城镇新增就业6.5万人，城镇登记失业率控制在3.1%以内，农村劳动力转移就业10.42万人，创建充分就业社区140个。率先在全省建立全民参保数据库，跨省异地就医信息网络实现互联互通。城市和农村低保分别从511元/月、3092元/年提高到566元/月、3570元/年。棚改大会战成效明显，实施棚户区改造4.5万户，建成保障房4.8万套，完成农村危房改造11534户，完成小康房建设2500户。“十件实事”全面完成。

公共服务持续提升。“全面改薄”工程扎实推进，持续推动教育资源向贫困地区、农村学校和薄弱学校倾斜，平坝、普定义务教育基本均衡发展工作通过国家评估验收，农村营养改善计划扩大到学前教育儿童。安顺学院改扩建工程顺利推进，安顺职院新校区建成投用。医药卫生体制改革深入实施，全面取消公立医院药品加成，启动多形式医联体建设和家庭医生签约服务。市人民医院实现整体搬迁，市妇幼保健院（市儿童医院）成功创建“三甲”医院，基层医疗卫生服务能力三年提升计划顺利实施，市县乡村四级医疗卫生机构疾病诊断、疾病治疗、康复护理、公共卫生服务和管理能力明显提升。公共文化服务水平不断提高，多彩贵州“广电云”户户用工程入网10万户，成功举办全市第一届文艺调演，以“双阅读”为载体的全民阅读活动广泛开展，虹湖音乐季、美丽乡村游成为特色文化惠民品牌，市奥体中心全面对外开放，全民健身活动蓬勃开展，人民群众精神文化生活不断丰富。

社会治理持续强化。创新社会治理，推动城乡基层民主建设，完成第十届村（居）委换届选举，92个城市社区达到国家建设标准，873个村建成农村社区服务站。覆盖城乡的法律服务网络不断完善，基层法律服务供给能力不断提升。依法分类处理信访诉求，矛盾纠纷和信访问题有效化解。强化社会治安综合治理，人民群众安全感和满意度逐年提升。安全生产和食品药品安全形势总体稳定。民族宗教、外事侨务、史志档案、粮食安全、人防、气象、老龄等各项工作取得新进展。

（七）坚持抓作风重实干，行政效能取得新提升

依法行政深入落实。坚持依法行政，严格执行规范性文件合法性审查和备案审查工作，依法行政制度体系不断完善。配合做好《安顺市虹山湖公园管理条例》等地方立法工作。自觉接受市人大的法律监督、工作监督和市政协的民主监督，办理人大代表建议85件、政协提案191件，办复率100%，认真听取各民主党派、工商联和无党派人士的意见建议。积极支持工会、共青团、妇联、文联、科协、残联、侨联、红十字会、计生协会等依法按章履行职责，各项工作不断取得新成效。

行政效能不断提升。“放管服”改革深入推进，“双随机、一公开”全面推开，公布实施新一轮权力清单和责任清单，行政审批事项减少到244项，取消行政审批中介服务事项12项，办理时限在法定时限基础上压缩了50％。政府信息公开工作扎实推进，电子政务加快建设，公共资源交易不断规范，政务服务持续向乡村延伸，服务群众“最后一公里”进一步畅通。

廉政建设扎实推进。全面落实中央八项规定、省委十项规定和市委实施意见，严格控制“三公”经费支出，全面彻底执行公务活动“禁酒令”。严格执行党风廉政建设责任制，深入推进干部作风建设，切实做到勤政、廉洁、务实、高效。加强行政监察和审计监督，加大反腐倡廉力度，从源头上防治腐败，持续营造风清气正的政务环境。

回顾过去一年的工作，我们深切体会到，在经济下行压力加大的情况下，我市能够实现持续快速发展，成绩来之不易！这是省委、省政府和市委坚强领导的结果，是市人大依法监督和市政协民主监督的结果，是全市各族人民团结拼搏、锐意进取的结果。在此，我谨代表市人民政府，向全市各族人民，向各位代表、政协委员，向各民主党派、工商联、无党派人士、人民团体，向驻安人民解放军、武警消防官兵、公安政法干警、中央和省驻安单位，向老同志及所有关心、支持我市改革发展和现代化建设的各界人士，表示衷心的感谢并致以崇高的敬意！

各位代表，在看到成绩的同时，我们也清醒地认识到存在的问题和不足。主要表现在：经济总量小、综合实力弱，实现全面小康任务艰巨；贫困面大、贫困人口多、贫困程度深，决胜脱贫攻坚任务重；产业基础薄弱、优势不突出，支撑经济发展的动力不强；生态环境比较脆弱，环境保护压力大；民生领域短板较多，公共服务保障能力不足；政府自身建设还存在薄弱环节，少数干部执行力、落实力不强，工作作风仍需进一步改进。为此，我们将正视存在的问题，采取有效措施，切实加以解决，决不辜负人大代表、政协委员的信任，决不辜负全市人民的重托！

二、2018年重点工作

2018年是贯彻党的十九大精神的开局之年，是改革开放40周年，是决胜全面建成小康社会、实施“十三五”规划承上启下的关键一年。做好今年的各项工作意义重大，使命光荣。

今年政府工作的总体要求是：全面贯彻党的十九大精神和习近平总书记在贵州省代表团重要讲话精神，以习近平新时代中国特色社会主义思想为指导，统筹推进“五位一体”总体布局和协调推进“四个全面”战略布局，坚持稳中求进工作总基调，坚持新发展理念，按照高质量发展要求，以供给侧结构性改革为主线，守好发展和生态两条底线，坚持主基调主战略，全力实施大扶贫、大数据、大生态三大战略行动，持续打好“一二四”攻坚战，决战脱贫攻坚、决胜全面小康，奋力续写新时代富美安顺加快发展新篇章！

今年全市经济社会发展的主要预期目标是：地区生产总值增长12%，500万元以上固定资产投资增长20%，规模以上工业增加值增长12%，社会消费品零售总额增长13%，一般公共预算收入增长10.6%，城乡居民人均可支配收入分别增长10%和12%，居民消费价格涨幅控制在3%以内。

围绕实现以上目标，我们将重点抓好七个方面工作。

（一）以脱贫攻坚为统领，全面抓实大扶贫战略行动

聚焦产业扶贫主攻方向。以农业园区为平台，积极推进产业扶贫和农业发展有机结合，大力调整农业产业结构，在确保粮食安全的前提下，全面调减玉米种植，重点发展蔬菜、食用菌、茶叶、烤烟、精品水果、中药材、生态畜禽、淡水生态养殖等特色优势扶贫产业。加快冷链物流设施建设，加强“农校”“农超”“农企”对接，大力发展农村电商，组织到珠三角、长三角、青岛等地开展农产品推介和销售。全力推进旅游扶贫“十大工程”，大力发展乡村旅游扶贫。创新产业扶贫项目与贫困农户的利益联结机制，推广“塘约经验”“一村一公司”“菜单式”扶贫等模式，支持贫困村抱团发展，统筹用好产业扶贫子基金，做大产业规模。

打好脱贫攻坚组合拳。瞄准特殊贫困人口精准帮扶，把扶贫和扶志、扶智结合起来，激发贫困人口内生脱贫动力，提高脱贫质量。聚焦深度贫困地区脱贫攻坚主战场，扎实抓好紫云深度贫困县，简嘎、大营两个极贫乡镇和178个深度贫困村脱贫攻坚工作。加大力度推进易地扶贫搬迁，做好2017年度剩余8个安置点项目收尾工作，2018年6月底全部实现搬迁入住。以深度贫困地区和自然村(寨)整体搬迁为重点，坚持城镇化集中安置和以县为单位集中建设，抓好2018年度易地扶贫搬迁工作。按照“四好农村路”要求，深入推进农村“组组通”公路三年大决战。落实教育扶贫资助减免政策，巩固提高“四重医疗保障”水平，大力实施农村危房改造。扎实推进东西部扶贫协作和社会帮扶，深入开展“千企帮千村”行动。对照“一达标两不愁三保障”要求，狠抓“两率一度”，实现西秀、平坝通过国家脱贫攻坚第三方评估验收正式退出贫困县，扎实做好普定、镇宁按国定标准退出贫困县工作。

大力实施乡村振兴战略。按照产业兴旺、生态宜居、乡风文明、治理有效、生活富裕的总要求，健全城乡融合发展体制机制，大力推动乡村振兴。以产业振兴为重点，积极发展高效设施农业和生态休闲观光农业，加快发展林下经济，培育壮大新型农业经营主体，推动要素跨界配置和农产品生产、加工、销售及休闲等相关服务业的有机整合，促进农村一二三产紧密融合、协同发展。持续深化“三权”促“三变”改革，有效激发农业农村发展活力。全面加快“四在农家·美丽乡村”建设，着力打造“八个小康行动计划”升级版，新建20个美丽乡村。加强农村基层基础工作，建立村务监督委员会，健全自治、法治、德治相结合的乡村治理体系。加强农村人才培训，培养造就一支懂农业、爱农村、爱农民的“三农”工作队伍。加强村落文化挖掘、保护、利用，注重文脉传承，提升乡风文明程度。

（二）以军民融合为突破，全面推进新型工业化建设

全力深化军民融合发展。贯彻落实《关于推动国防科技工业军民融合深度发展的意见》，完善军民协同机制，加强人才交流和培养，多途径、多领域促进军民融合深度发展。依托安顺高新区、民用航空产业国家高技术产业基地、国家通用航空产业综合示范区等平台，聚焦航空制造、通用航空、航空服务等产业，全力打造军民融合产业发展集聚区。加快军民融合产业项目建设，全力推动贵飞工业联合体签约项目落地，实现新扬复合材料正式投产，建成安大航空锻造产业园锻造生产线，完成安吉精铸航空产业园一期建设，完成安捷航宇公司组建运营，力争启动建设8万辆乘用车新能源汽车城项目、云马5000辆新能源客车升级改造项目，做好贵飞民用无人机、风雷科技高低压氧舱、新能源汽车产品推广应用。积极组织参加全省军民融合博览会，做好第二届爱飞客通用航空飞行大会筹备工作。积极推进“三线文化”开发利用，以黎阳航空小镇、云马旅游产业园建设为抓手，推动军民文化融合与产业发展相互促进，齐头并进。

全力推动新旧动能转换。以“千企改造”工程为抓手，振兴实体经济，促进产业高端化、绿色化、集约化发展。实施打磨冲、桐鑫、安发、盘龙树等煤矿技改建设，全市煤炭产量达到800万吨。完成安顺电厂机组供热项目建设，全年供汽45万蒸吨以上。建成红星发展年产1万吨硝酸钡和年产3万吨硫酸钡二期扩建项目。建成安顺铝业产能等量置换技改项目，引进铝型材、铝板材生产线，支持安顺铝业与锦程科技、安吉精铸等企业开展上下游产业链合作。大力发展以百灵制药为龙头的特色民族医药产业，支持大健康医药物流中心项目建设，加快安酒、平坝窖酒、黄果树酒等技改步伐和产能提升，培育壮大特色食品、手工艺品、日用快消品等产业。推进中国（安顺）石材城市场化运作，建立贵州石材商品交易中心，打造石材线上线下交易平台，办好第四届石博会，促进石材产业聚集发展。加快数据“聚通用”，抓好国家大数据综合试验区安顺示范园和“云上贵州·安顺彩云”系统平台建设。加快推进西部生态智谷和西秀智能终端产业园建设，培育和引进一批大数据、云计算、物联网、高端软件服务、人工智能等优强企业。推进大数据与实体经济深度融合，开展“万企融合”大行动，打造一批融合标杆项目，建设一批融合示范项目。

全力增强企业发展活力。深化园区体制机制改革，提升园区承载力，促进关联企业聚集发展，西秀产业园区产值突破300亿元、民用航空国家高技术产业基地产值突破200亿元、安顺高新区产值突破190亿元。抓好“双服务”“百千万”工程，实行清单式、精细化调度帮扶，新增入库企业45户以上。搭建好政银企合作平台，用好用活“中小企业创业贷”、“贵工贷”、中小企业应急周转金、煤炭转型升级发展资金等帮助企业解决融资难题。提升企业科技创新能力，制定和完善科技创新平台激励机制和办法，促进贵飞无人机工程技术研究中心等31家创新平台发挥行业科技支撑作用，支持引导安大公司、新安公司等有条件的企业建设研发机构、检验检测中心，加快形成自主知识产权和核心竞争力。支持企业开拓市场，组织参加国际航空航天展、国际石材展、广交会、西博会、酒博会、数博会等国内外大型展会，提高产品知名度和市场占有率。

（三）以全域旅游为龙头，全面提升现代服务业水平

积极推进全域旅游加快发展。加快旅游综合交通枢纽和配套服务设施建设，深化“旅游+”多产业融合模式，构建全域旅游大格局，成功创建全域旅游示范区。重构城市旅游功能格局，升级城市旅游产品业态，塑造城市旅游整体形象，全力打造贵州旅游名城。用好旅游资源大普查成果，重点打造休闲度假、康体养生、户外运动、科普探险等一批新产品、新业态，启动建设安顺“国家路游公园”，加快建设生态体育公园。加大旅游市场主体培育力度，加快黄果树旅游集团公司主板上市步伐，吸引优强企业参与景区旅游开发。推进黄果树·屯堡申报世界自然与文化双遗产工作。继续办好黄果树瀑布节、格凸河国际攀岩、坝陵河国际跳伞等节庆赛事活动，提升镇宁黄果树国际半程马拉松为全程马拉松。支持紫云办好第五届安顺旅游产业发展大会。全年接待游客9490万人次，增长30%，实现旅游总收入990亿元，增长32%。

积极推进现代服务业繁荣发展。实施现代服务业“十百千”工程，培育1个省级现代服务业发展示范区、2个聚集区和一批重点项目，推进大兴东产业园建成投用，力争设立大兴东跨境电商，加快黔中（安顺）物流园区发展，全面推进黄铺新区建设，支持澳维天下乐城实现营运。实施新一轮大健康产业六项行动计划，办好贵州省第四届大健康医药产业发展大会。支持社会力量提供教育、文化、养老、医疗等专业化、综合性服务，积极发展电子商务、快递物流、金融服务、智能家居、健康养生、文化创意、数字经济、共享经济等现代服务业。全年实现服务业增加值465亿元，增长14%。

积极推进消费快速增长。制定完善消费政策，大力发展新兴消费，稳步扩大大众消费，逐步发展高端消费，探索定制个性消费。开展质量提升行动，引导企业增品种、提品质、创品牌，增加高品质产品消费，推进“三品一标”工作，创建6个以上贵州省名牌产品，争创1个省长质量奖。扶持会展业加快发展，鼓励举办汽车、家居、服装、农产品等各类博览会、展销会，加大批零住餐企业培育，支持山里江南、苗岭屯堡、多彩万象旅游城等成为新的消费增长点。整顿和规范市场秩序，健全从生产到消费全过程监管体系，加强消费者权益保护。

（四）以“新型城镇化+”为主线，全面统筹城乡融合发展

全力加快试点工作。全面推进“一分三向”新型城镇化安顺模式，加快建立多元化可持续的城镇化投融资机制、城镇建设用地增加规模同吸纳农业转移人口落户数量挂钩机制，深化户籍制度改革，落实“1+13”文件体系，加快农业转移人口市民化进程，常住人口城镇化率达到54%，户籍人口城镇化率达到41%。深入推进“多规合一”，加强城市设计工作，扎实推进海绵城市、地下综合管廊、轨道水道慢道“三道”示范工程建设，实施城市“双修”试点项目。

全力完善基础设施。加快黄果树机场改扩建工程和乐平通用机场建设，加大航线培育力度，力争开通安顺至成都航线。加快推进安六铁路建设。开工建设都香（安顺段）、乌当至长顺（安顺段）高速公路，加快建设紫望高速公路，建成普织高速公路。加快普通国省道干线升级改造，积极推进农村公路新改建工程。开工建设龙井湾、核桃冲、朵贝等一批水库，加快推进黄家湾、大洞口、坝陵河、木桥等水库建设，建成红坪、木拱河、小鲁灰、雨棚、芦坝等水库。推进省部共建水利精准扶贫示范区建设，开工建设25个典型示范项目。加快新一轮农村电网改造升级，完成电力基础设施投资5.16亿元。大力推进城镇燃气基础设施建设，开展乡镇管道燃气供应试点。加快4G网络建设，推动5G通信发展，全市城区及重点乡镇实现免费WiFi全覆盖，城域网上链带宽达到600Gbps以上，完成信息基础设施投资12亿元。

全力统筹城乡建设。实施《安顺市城市总体规划（2016-2030年）》。开工建设现代有轨电车3、4号线一期工程。启动建设主城区南环线，打通一批断头路，实施东出口改造工程。完成主城区供水三期工程。加快推进历史文化街区、娄湖片区等棚户区、城中村改造建设，不断丰富安顺高铁新城、虹山湖新区城市内涵。开工建设观安路等与贵安新区互联互通道路、黄桶至丁旗大道、紫云至猫营快速干道，加快建设平坝东外环线、贵安大道（经开区至黄果树段），建成关花大道。加快平坝高铁新区、普定新城、镇宁环翠、关岭顶云、紫云新城建设。引导特色小城镇健康发展，力争新增5个示范小城镇。完成全市所有行政村村庄规划编制，完成重点区域村土地利用规划编制和其他区域村允许建设区划定，推进田园综合体建设。做好第三次全国土地调查工作。支持经开区办好第八届安顺市新型城镇化推进暨小城镇建设发展大会。

（五）以绿色发展为引领，全面加快美丽安顺建设

全面加快生态建设。深入实施生态修复工程，构建生态廊道和生物多样性保护网络，完成生态保护红线、永久基本农田、城镇开发边界三条控制线划定工作。开展大规模国土绿化，加强天然林保护，完成退耕还林还草14.93万亩，综合治理石漠化面积102平方公里，治理水土流失面积220平方公里，森林覆盖率达到56%。实施“路池生态系统”工程建设，加快邢江河、虹山湖等湿地公园建设。推进城镇公共绿地、环城林带、交通沿线及景区景点景观绿化建设，建成综合公园、山体公园、社区公园、街头游园（广场）34个。推进国家森林城市创建工作，所有建制县区成功创建省级森林城市。

全面加强环境治理。实行最严格的生态环境保护制度，依法严处各类环境违法行为，深入实施大气、水、土壤污染治理三大行动计划。全面整改中央环保督察反馈的问题，确保各类环保基础设施正常运行，进一步改善环境质量。严格落实河长制，加大饮用水源保护区等重点区域保护力度。严格耕地保护，加强地质灾害防治。坚持不懈推进“厕所革命”。完善城乡垃圾处理、污水处理基础设施，新建改造垃圾处理和收运设施10个，新增雨污分流管网100公里以上，完成镇宁、关岭、紫云县城污水处理厂提标升级。加强农业面源污染治理，提高秸秆综合利用率。县级以上城市污水处理率、城乡垃圾无害化处理率分别达到93%、89%，县城以上城市空气质量优良天数比例达到95%以上。

全面加大节能减排。加强重点用能和重点排放单位监管，支持节能降耗技术改造，禁止新增高能耗、高排放项目。优化用能结构，加快太阳能、风能、生物能等清洁能源推广使用步伐，推进“煤改电”“煤改气”。能源消费增量、单位地区生产总值能耗、化学需氧量、氨氮排放量等指标控制在省下达标准范围内。

（六）以改革开放为引擎，全面增强发展活力

聚焦问题抓改革。推进供给侧结构性改革，提高全要素生产率，努力培育和形成新供给、新动力。继续深化财税体制改革，降低企业成本。深化金融改革，有效防范金融风险，增强金融服务实体经济能力，促进金融和经济良性循环、健康发展。加强国资监管，提升平台公司经营管理能力，实现国有资产保值增值。大力实施企业上市“百千行动计划”，积极发展股权融资、债券融资。全面推进不动产统一登记制度和市场主体“多证合一”登记制度改革。大力发展民营经济，实施百亿民营企业培育工程，开展中小企业“星光”行动，民营经济增加值占生产总值比重达到66%以上。推进城市公交系统改革，实现公交车全部公营。推进农业保险试点，促进农业保险扩面、增品、提标。继续推进平坝农村集体产权制度改革、普定自然资源统一确权登记两个国家级试点工作。实施创新驱动发展战略，鼓励贵飞工业联合体、西秀智能终端创新联盟等构建多形式、多渠道创新合作机制。综合科技进步水平指数达到60%，争创国家创新型城市。

夯实平台促开放。继续推进以国家新型城镇化综合试点为引领的“1+5”开放平台建设，推动安顺高新区加快发展，聚集一批航空航天、装备制造、新医药、电子信息等高新企业，推动安顺经济技术开发区升格国家级开发区工作。巩固国家卫生城市、国家园林城市创建成果，继续开展全国文明城市创建工作。深化与贵阳、贵安新区的区域合作，进一步加强与青岛的扶贫协作，积极参与“一带一路”倡议三年行动，主动融入长江经济带、珠江—西江经济带发展，深化与港澳台沟通对接，加强与越南、泰国、缅甸等东盟国家和德国、法国、瑞士等欧洲国家交流合作。完善安顺公用型保税仓库功能建设，提高保税仓储成交量。实施外贸进出口翻番计划，确保进出口总额增长15%，实际利用外资增长20%。

多措并举强招商。深入实施“千企引进”工程，组织开展“产业大招商突破年”活动，围绕脱贫攻坚、军民融合、通用航空、智能制造、全域旅游等领域，紧盯500强企业和上市公司，引进一批具有较强辐射带动作用的重大项目。在市权范围内制定完善招商引资优惠政策，吸引更多企业在安投资兴业。进一步提升服务效能，优化营商环境，加快推动项目落地。全年确保实现新增项目数、新增项目合同投资总额、新增到位资金增长25%，新增项目开工数、当年投产项目数增长20%。

（七）以改善民生为根本，全面提升群众获得感

着力强化社会保障。推进就业创业促进工程，实施“雁归兴安”“锦绣计划”“千名大学生创业行动计划”。加快建设城乡、区域、群体发展平衡、制度完备衔接的社保体系，全市基本社会保险覆盖率达到90%。加快建立健全多主体供给、多渠道保障、租购并举的住房制度，建成各类保障性住房3.2万套，公租房分配率达到95%以上。完善社会救助体系，建立社会捐赠、群众互助等多形式的救助机制，提升社会救助水平。

着力发展社会事业。大力实施教育提升工程，缩小城乡教育差距，推进各级各类教育全面协调发展，所有县（区）实现县域义务教育发展基本均衡。加快推进市一中改扩建工程、安顺学院改扩建二期工程，支持安顺学院开展“双一流”建设、安顺职院开展优质高职院校建设。加快建设健康安顺，深化医药卫生体制改革，建立完善分级诊疗、现代医院管理、药品供应保障、全民医保和综合监管制度，推进计生服务管理改革。深入推进“百院大战”工程，建成市公共卫生服务中心。增强文化自觉和文化自信，培育和践行社会主义核心价值观，加强思想道德建设，办好新时代农民（市民）讲习所。推动文化事业和文化产业加快发展，深入实施文化惠民工程，加强文物保护利用和文化遗产保护传承，繁荣发展黔中文化。支持文学文艺创作，开展政府文艺奖评选活动，丰富“全民阅读·书香安顺”系列活动开展形式。整合利用现有资源，加快建设老年活动中心等公共文化服务设施。发展体育事业和体育产业，培育建设体育特色小镇，积极备战全省第十届运动会和全省第九届少数民族传统体育运动会，办好第二届全市运动会，广泛开展全民健身活动。加强国防后备力量建设，完成新一轮省级双拥模范城创建申报工作。加快发展慈善、老龄、残疾人事业，进一步做好国防教育、民族宗教、新闻出版、广播影视、防灾减灾、史志、文物、档案、外事、侨务、对台等工作。

着力推进社会治理。打造共建共治共享的社会治理格局，加强社会治理制度建设，提高社会治理社会化、法治化、智能化、专业化水平。加强社区治理体系建设，完善社区配套设施和服务功能，构建和谐社区。推进安顺经开区农村社区治理实验区建设。严格落实安全生产责任制，坚决遏制重特大安全事故发生。继续加强食品药品安全监管，着力提升技术保障能力和水平。强化矛盾纠纷排查调处，全力推动阳光信访、法治信访、责任信访。加快社会治安防控体系建设，依法打击和惩治黄赌毒黑拐骗等违法犯罪活动，保护人民人身权、财产权、人格权，持续提升人民群众的安全感和满意度。

各位代表，民生是为政之本。围绕保障和改善民生，我们既尽力而为，又量力而行，一件接着一件办，一年接着一年干。今年将继续办好十件实事：①减少农村扶贫对象8万人；实施易地扶贫搬迁1.5万人。②城镇新增就业5.5万人，新增创业3000户，新增创业带动就业1.1万人。③城镇和农村居民最低生活保障标准在2017年基础上分别提高5%和8%。④完成农村公路安全生命防护工程1000公里；建成农村通组路1500公里。⑤完成20万人农村饮水安全巩固提升工程。⑥建成农村义务教育学校标准化食堂89个；开展教育精准扶贫资助贫困学生1.5万人。⑦完成10所公立医院改扩建；实施260个村卫生计生室改扩建；培训基层医疗卫生人员2000人，实现村医培训全覆盖。⑧完成棚户区改造3万户，完成农村危房改造11453户。⑨新增主城区公共停车位1000个；升级改造13个农贸市场。⑩完成“天网”四期工程，建成视频监控点、卡口1642个。

三、扎实有效推进政府自身建设

各位代表，新时代、新战略、新目标、新任务对政府工作提出了新的更高要求。我们必须切实增强新时代意识，进一步解放思想、开拓创新，更加高度自觉、更加扎实有效推进政府自身建设。

一是提高政治站位。深入学习贯彻党的十九大精神和习近平总书记在贵州省代表团重要讲话精神，以习近平新时代中国特色社会主义思想为指导，增强“四个意识”，树牢“四个自信”。把政治建设摆在首位，继续推进“两学一做”学习教育常态化、制度化，抓好“不忘初心、牢记使命”主题教育，切实推进政府系统全面从严治党，旗帜鲜明讲政治，凝心聚力抓发展。

二是推进依法行政。严格践行法无授权不可为、法定职责必须为的执政理念，用法治思维实施政府管理。健全完善依法决策机制，把公众参与、专家论证、风险评估、合法性审查、集体讨论决定确定为重大行政决策法定程序，进一步提高决策科学化、民主化、法治化水平。加强与民主党派、工商联、无党派人士、人民团体的沟通协商，深化人大代表议案建议和政协提案的办理联系机制。重视司法、舆论、社会监督，真正做到决策听民意、执行察民情、效果听民声。

三是持续改进作风。大力弘扬“团结奋进、拼搏创新、苦干实干、后发赶超”的新时代贵州精神，坚持一切为人民打算、共同为小康实干。大兴调查研究之风，大兴真抓实干之风，大兴勤俭节约之风，深入基层一线，研究解决问题，勇于担当、主动作为，发扬“钉钉子”精神，不断提高执行力和落实力。深入推进“互联网+政务服务”，加快市县乡村政务服务平台标准化建设，大力整治庸政懒政怠政现象，不断提高行政效能。

四是狠抓廉政建设。坚持以上率下、持廉守正，依法规范行使行政权力，干干净净为人民做事。认真贯彻中央八项规定实施细则，坚持不懈纠正“四风”，做到真管真严、敢管敢严、长管长严。配合推进国家监察体制改革，实现对所有行使公权力的公职人员监察全覆盖。继续完善审计监督、民生监督机制。严厉整治各种违纪违法行为，强化不敢腐的震慑，扎牢不能腐的笼子，增强不想腐的自觉，把党风廉政建设和反腐败工作不断引向深入。

各位代表，中国特色社会主义进入新时代，全面建成小康社会步入决胜期，富美安顺正在书写新篇章。让我们更加紧密地团结在以习近平同志为核心的党中央周围，在市委的坚强领导下，紧紧依靠全市各族人民，不忘初心、牢记使命，感恩奋进、砥砺前行，以永不懈怠的精神状态和一往无前的奋斗姿态，为加快建设新时代富美新安顺而努力奋斗！
